# DigiPatICS: Digital Pathology Transformation of the Catalan Health Institute Network of 8 Hospitals—Planification, Implementation, and Preliminary Results

**DOI:** 10.3390/diagnostics12040852

**Published:** 2022-03-30

**Authors:** Jordi Temprana-Salvador, Pablo López-García, Josep Castellví Vives, Lluís de Haro, Eudald Ballesta, Matias Rojas Abusleme, Miquel Arrufat, Ferran Marques, Josep R. Casas, Carlos Gallego, Laura Pons, José Luis Mate, Pedro Luis Fernández, Eugeni López-Bonet, Ramon Bosch, Salomé Martínez, Santiago Ramón y Cajal, Xavier Matias-Guiu

**Affiliations:** 1 Department of Pathology, Vall d’Hebron University Hospital, CIBERONC, 08035 Barcelona, Spain; joscastellvi@vhebron.net (J.C.V.); sramon@vhebron.net (S.R.y.C.); 2 Functional Competence Center, Information Systems, Catalan Health Institute (Institut Català de la Salut), 08006 Barcelona, Spain; plopezg@gencat.cat (P.L.-G.); ldeharo@gencat.cat (L.d.H.); eballesta@gencat.cat (E.B.); 3 Center for Telecommunications and Information Technology (Centre de Telecomunicacions i Tecnologies de la Informació, CTTI), Catalan Health Institute (Institut Català de la Salut), 08006 Barcelona, Spain; mrojasabusleme@gencat.cat; 4 Economic and Financial Management, Catalan Health Institute (Institut Català de la Salut), 08006 Barcelona, Spain; miquel.arrufat@gencat.cat; 5 Image Processing Group, Technical University of Catalonia (UPC), 08034 Barcelona, Spain; ferran.marques@upc.edu (F.M.); josep.ramon.casas@upc.edu (J.R.C.); 6 Digital Medical Imaging System of Catalonia (SIMDCAT), TIC Salut, 08005 Barcelona, Spain; cgallego@ticsalutsocial.cat; 7 Department of Pathology, Germans Trias i Pujol University Hospital, 08916 Badalona, Spain; lponsmar.germanstrias@gencat.cat (L.P.); jlmate.germanstrias@gencat.cat (J.L.M.); plfernandez.germanstrias@gencat.cat (P.L.F.); 8 Department of Pathology, Doctor Josep Trueta Hospital of Girona, 17007 Girona, Spain; elopezbonet.girona.ics@gencat.cat; 9 Department of Pathology, Verge de la Cinta Hospital of Tortosa, 43500 Tarragona, Spain; rbosch.ebre.ics@gencat.cat; 10 Department of Pathology, Joan XXIII University Hospital of Tarragona, 43005 Tarragona, Spain; mgonzalez.hj23.ics@gencat.cat; 11 Department of Pathology, Arnau de Vilanova University Hospital, 25198 Lleida, Spain; xmatias@bellvitgehospital.cat; 12 Department of Pathology, Bellvitge University Hospital, CIBERONC, 08907 Barcelona, Spain

**Keywords:** digital pathology, computational pathology, artificial intelligence, deep learning, implementation, workflow, primary diagnosis, LIS, telepathology, network

## Abstract

Complete digital pathology transformation for primary histopathological diagnosis is a challenging yet rewarding endeavor. Its advantages are clear with more efficient workflows, but there are many technical and functional difficulties to be faced. The Catalan Health Institute (ICS) has started its DigiPatICS project, aiming to deploy digital pathology in an integrative, holistic, and comprehensive way within a network of 8 hospitals, over 168 pathologists, and over 1 million slides each year. We describe the bidding process and the careful planning that was required, followed by swift implementation in stages. The purpose of the DigiPatICS project is to increase patient safety and quality of care, improving diagnosis and the efficiency of processes in the pathological anatomy departments of the ICS through process improvement, digital pathology, and artificial intelligence tools.

## 1. Introduction

**Figure 1:**
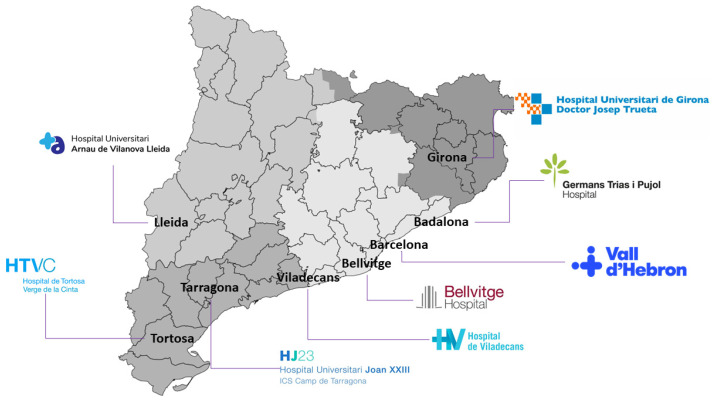
Map of Catalonia and the 8 ICS hospitals.

The Catalan Health Institute (Institut Català de la Salut, ICS) is the largest provider for the Catalan Health Service, the insurer of universal health coverage in Catalonia. It is the company with the most employees in Catalonia and the largest public company in Spain with almost 39,000 professionals who provide services to almost six million people throughout the territory [1]. The ICS manages 283 primary care teams, three large high-tech tertiary hospitals (Vall d’Hebron, Bellvitge, and Germans Trias), four regional reference hospitals (Arnau de Vilanova in Lleida, Joan XXIII in Tarragona, Josep Trueta in Girona, and Verge de la Cinta in Tortosa), and a regional hospital (Viladecans) (Figure 1). The ICS accounts for 7% of the Catalonian government budget with over 40 million primary visits and over 100,000 surgical interventions yearly.

Some laboratories are beginning to deploy successfully digital pathology solutions for routine diagnosis which we believe will be a growing trend in the next few years [2,3,4,5,6,7,8,9,10]. In Catalonia, the DigiPatICS project plans to accomplish a complete digital pathology transformation of primary histopathological diagnosis for over 168 pathologists. Many groups have reported equivalency between digital pathology and conventional pathology [11,12,13,14,15,16,17,18,19,20,21,22,23,24,25,26,27].

With the DigiPatICS project, we aim to increase patient safety and quality of care, improving diagnosis and the efficiency of processes in Pathological Anatomy departments of the ICS through digital pathology and artificial intelligence (AI) tools [28]. With digital pathology, we aim for a network of eight hospitals to work as one in terms of case sharing and teaching, putting all our patients on equal footing. This transversal digital transformation will have an impact on the care of patients treated by all medical and surgical specialists.

First, we created a network between ICS centers. This helped us increase the reproducibility and quality of diagnoses, as well as offered greater equity and safety to patients. In turn, this network approach facilitated remote diagnosis, case sharing, subspecialization, and teaching for pathologists. In addition, we aimed for better working conditions, impacting the optimization of workflows, productivity, and, finally, turnaround times. We also intended to improve ergonomics and postural health, as well as to facilitate morphometric tools and the quantification of diagnostic and prognostic biomarkers to involve the optimization of time and a higher quality in diagnosis.

From a more technical point of view, the aim was to achieve a central digital repository of images on the network, thereby reducing the burden of slide file management and integrating medical imaging with SIMDCAT, a digital medical imaging system used in Catalonia. It was also intended as a subproject to establish bidirectional communications with other locations, such as operating rooms.

The project included the development of artificial intelligence tools with machine learning and deep learning, taking advantage of the availability of whole slide images (WSIs) that were obtained after digitization. The objectives were to recognize tissue patterns, select tumor areas, and quantify them, among others. Hopefully, this use of artificial intelligence tools will contribute to improving the quality of diagnosis and the efficiency of processes.

## 2. Materials and Methods

DigiPatICS was created as a European Regional Development Fund (ERDF) project with European funds for the optimization of anatomopathological diagnosis in a network of public ICS hospitals in Catalonia through digitalization and artificial intelligence tools.

Subsequently, a market consultation was carried out, and, finally, it was tendered with the expedient CSE/CC00/1101202869/20/AMUP [29].

### 2.1. Planning, Scope, and Tender Process

A definition of needs was then carried out. We firmly believe meticulous planning is essential, taking into account all functional and technological requisites. Failing to detail such requirements can end in the failure of a digitization project, resulting in expensive scanners installed in pathology laboratories that are barely used. It is also important to highlight that going digital is not about acquiring pathology scanners; we focused our project on the purchase of a service with a shared risk with the bidder to achieve our objectives. Since this transformation was meant to be a one-way street with no possibility of going back to microscopes, all planning needed to included sufficient contingencies to avoid any kind of downtime for pathologists, as well as benefiting from the potential added value.

The purpose of the DigiPatICS project was to increase patient safety and quality of care, improving diagnosis and the efficiency of processes in pathological anatomy departments of the ICS using digital pathology and artificial intelligence tools.

In defining the scope, several questions arose:

- Do we want to save the whole slide images (WSIs) forever? Who will store them?
- Is our laboratory information system (LIS) ready?
- Do we want to (or have to) address pre-analytics?
- Do we want to address dark-field microscopy (direct fluorescence, FISH)?
- Do we want to digitize the macroscopic images?
- Do we need to update the hospital network?
- Do we need to update our pathologists’ workstations?
- Do we want to share cases with the outside world?
- Is teaching important?
- Do we want artificial intelligence (AI) algorithms?
- Do we want to do telepathology?
- What do we do with cytology?
- Do we have money for everything?

Those concerns and how they were resolved will be addressed in the next pages, but we can already answer some of these. We did want to store all the WSIs forever and to use that repository to train our own artificial intelligence algorithms, which is clearly one of the great advantages of such a transformation. We also believed that this project must be an integral transformation, including routine histopathology, fluorescence, research, and macroscopic images. Tools for teaching and teleconsultation should be included, which meant having the option to share images outside our hospitals’ secured LAN. We realized that our laboratory information system (LIS), preanalytics, network, and pathologist workstations all needed substantial upgrades to be able to undertake such a transformation [9].

What about cytology? Digitizing cytology, even if feasible [30,31,32,33], has some particularities: scanning times can be much longer than in histology (due to the need for more resolution, larger scan area, zero tolerance of out-of-focus areas, and Z-stacking). That means needing to install more scanners to be able to take on the same activity, and it impacts storage needs. Dark-field scanning (FISH) has similar issues, but there was a significant difference in the volume of slides to scan. Cytology involves a large number of samples to digitize in our hospitals (over 400,000 each year), which is not affordable currently in this project due to budgetary constraints.

The activity addressed in our project included all bright-field, routine histopathology, histochemistry, immunohistochemistry, direct immunofluorescence, ISH, and FISH slides. Cytology was scanned on an as-needed basis.

In Table 1, we summarize the total number of slides generated during 2019 at our eight hospitals broken down by type. To that number of over 1 million slides, an expected growth in activity of 10% to 15% must be added each year. In addition, some resources were reserved for research and non-strictly routine samples and were not accounted for in these numbers.

Regarding the amount of personnel involved, DigiPatICS provided service to 107 pathologists, 7 biologists, 40 residents, and 14 observers, adding up to a total of 168 professionals working with digital diagnosis.

In the tender, all relevant aspects were taken into account for bidder evaluation, as shown in the following list:

**Award criteria** (***Total: 100 points***.)
  - Automatic evaluation criteria. (*51 points*)
    ○ Economic valuation. (*40 points*)
      ▪ Evaluation of the financial offer. (*30 points*)
      ▪ Evaluation of the maintenance offer. (*10 points*)
    ○ Automatic technical evaluation. (*11 points*)
      ▪ Quality management system. Certification of processes and algorithms. (*3 points*)
      ▪ Process consulting. (*1 point*)
      ▪ Image management platform adaptations. (*3 points*)
      ▪ Storage for research slides. (*1 point*)
      ▪ Short-term “hot” storage. (*3 points*)
  - Criteria subject to judgment value. (*49 points*)
    ○ Scanners: deployment and image quality. (*17 points*)
    ○ Diagnostic viewer. (*8 points*)
    ○ Image management platform. (*4 points*)
    ○ Training module. (*2 points*)
    ○ Built-in tools and algorithms. (*3 points*)
    ○ Architecture and monitoring. (*1 point*)
    ○ Definitive storage in SIMDCAT. (*4 points*)
    ○ Integration of case information in a unified model. (*2 points*)
    ○ Server infrastructure requirements and DPCs. Coherence, management model, virtualization. (*2 points*)
    ○ Workstations. (*2 points*)
    ○ Artificial intelligence. (*3 points*)
    ○ Implementation and additional improvements. (*1 point*)

However, some aspects were considered very difficult to assess by evaluating their technical characteristics alone, and that is why three technical tests were defined for the scanner, viewer, and image management platform.

**Figure 2:**
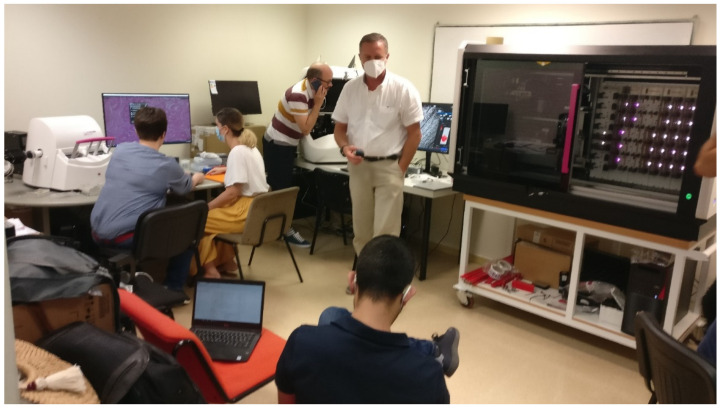
Testing room for test 1, showing the Palex 3DHISTECH team and equipment (PANNORAMIC SCAN II, PANNORAMIC 300 Flash DX, and PANNORAMIC 1000 Flash DX).

For the scanner test, a large sample of glass slides from the 8 hospitals was collected and fed to all the scanners offered by the 3 bidders for a week at 24 h a day. In the fastest scanner, over 10,000 glass slides were digitized. Real scanning speeds, jamming, incidents, etc. were recorded to ensure the reliability of the equipment (Figure 2).

**Figure 3:**
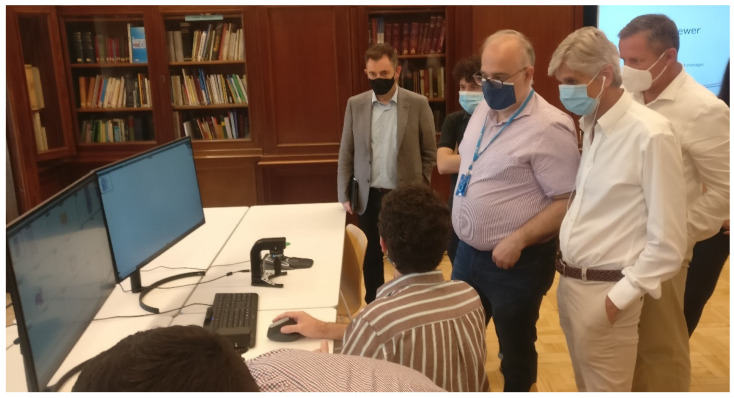
Testing room for tests 2–3, showing from left to right, Miquel Arrufat (ICS), Ramon Bosch (ICS), Pablo López-García (ICS), Josep Maria Argimon (ICS), and Tamás Regényi (3DHISTECH) with the Palex 3DHISTECH equipment (LG 32HL512D 8MP Diagnostic Monitor, Logitech MX Vertical Ergonomic Wireless Mouse, and 3DHISTECH SlideDriver).

In the second and third tests, we brought together a group of pathologists from all the involved hospitals, along with IT experts, and they assessed the functionalities of the viewing software and the image management platform, as well as the image quality offered by the scanners using images scanned from ICS samples during the first technical test (Figure 3).

The contract file was definitively awarded by the decision of the Managing Director of the Catalan Health Institute (ICS) to the Palex Medical, S.A. 3DHISTECH Digital Pathology solution. This solution stood out for the proposal in the following main aspects:

- **Scanners:** Technical requirements, deployment requirements, and technical service requirements.
- **Image Management Platform:** Diagnostic Viewer, learning platform, and quantification modules.
- **Architecture:** SIMDCAT interaction (DICOM), unified image management model (for all slide types), infrastructure management, and coherence model.

### 2.2. Scanners and Technology for Obtaining Whole Slide Images (WSIs)

Twenty-four scanners were installed and integrated into the workflow of the eight hospitals. Different scanner models were deployed according to the needs of each institution. In Table 2, we summarize the number of each scanner type and the capabilities.

The PANNORAMIC 1000 Flash DX (3DHISTECH Ltd., Budapest, Hungary) (P1000) is a large (154 × 100 × 91 cm) and heavy (270 kg) scanner, but it offers the largest slide capacity on the market at 1000 slides (using Leica slide racks, slide loading capacity could be further increased to 1200). It is the fastest whole-slide scanner on the market at up to 100 slides per hour and 2000 slides per day (at 40× resolution, 0.25 μm/pixel, single layer). The P1000 uses Sakura slide racks that seamlessly integrated with our laboratory workflow and allowed for priority slide handling and scanning in arbitrary order because it is flexible and automatic. It is also being used for double-width slides. Regarding image quality, it is able to scan at 0.25 μm/pixel, which is the 40× resolution equivalent (industry standard), and also at 0.12 μm/pixel, which is roughly the 80× resolution equivalent. Multilayer (Z-stack) and extended focus scanning are available, as well as automatic water immersion [34,35]. Furthermore, thanks to its AI-based software control, it is able to automatically rescan suboptimal slides, adding multilayer scanning if required. The P1000 are used for all bright-field and double-width slide related imaging.

The PANNORAMIC 300 Flash DX (3DHISTECH Ltd., Budapest, Hungary) (P300) is a fast bright-field and fluorescence scanner capable of high throughputs as a standalone machine in smaller institutions or serving as backup for P1000s in larger hospitals. It has the capacity for 300 slides, and its use is mainly for fluorescence, scanning FISH and direct immunofluorescence [36].

Both the PANNORAMIC SCAN II and the PANNORAMIC MIDI (3DHISTECH Ltd., Budapest, Hungary), with slide capacities of 150 and 12 slides, respectively, are mainly focused on fluorescence imaging but are still able to scan bright-field images, even though they are slower than their high-throughput counterparts [37].

### 2.3. Macroscopic Imaging

Regarding macroscopic imaging, 13 MacroPATH QX systems (Milestone Medical, Sorisole, Italy) were installed to obtain and incorporate gross imaging into the workflow. All the images were stored on the DigiPatICS servers and were fully integrated and available at the pathologist workstations for making diagnoses.

### 2.4. WSI Viewing: Hardware

**Figure 4:**
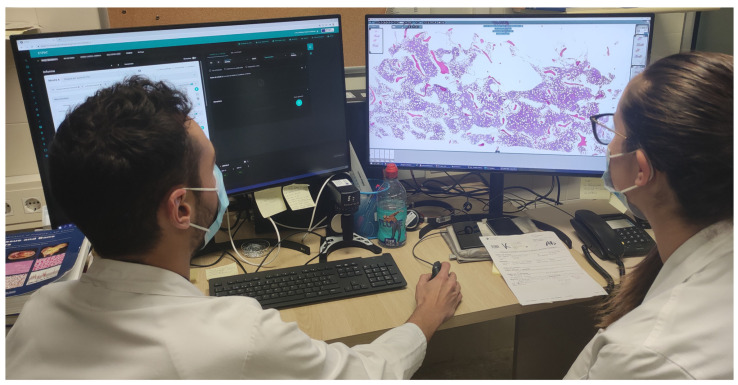
A pathologist in his office with a resident diagnosing with 2 LG 32HL512D 8MP diagnostic monitors.

To be able to view WSIs, 183 new workstations were installed (Table 3) for pathologists, residents, biologists, observers, and meeting rooms. Each pathologist workstation consisted of two 32-inch 4K UHD (3840 × 2160 pixel) diagnostic medical-grade FDA-approved monitors (LG 32HL512D) (Figure 4) that could be used indistinctly in flexible ways. Normal intended use is for a pathologist to have an LIS with all laboratory data, clinical data, and reporting available on one monitor, while on the other, a microscopic image is displayed. However, both monitors could be used for microscopic images, or both could be used for reporting, clinical data, bibliography, or other tasks. Biologist workstations were the same as those of the pathologists. Residents and observers shared the same workstations; however, they only consisted of one LG 32HL512D medical-grade monitor due to space constraints (the dual-monitor setup required over 150 cm of desk surface).

**Figure 5:**
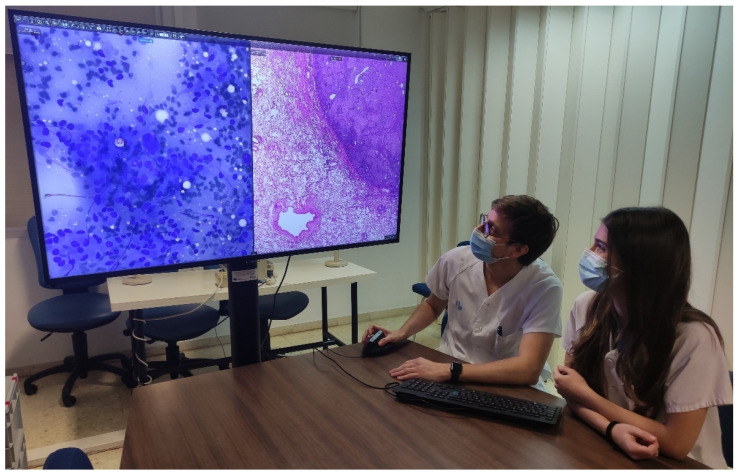
Residents diagnosing using a 55-inch 4K UHD monitor (55UH5F-B).

Thirteen additional workstations with six 55-inch 4K UHD monitors and seven 4K UHD DICOM ProBeam LG projectors were installed in small and medium meeting rooms for teaching and clinical sessions as a replacement for multi-head microscopes (Figure 5).

Each workstation was comprised of an Intel Core i5-9600K processor (Intel Corporation, Santa Clara, CA, USA), 16 Gb RAM, 512 Gb SSD, and an RTX 2060 graphics card (Nvidia Corporation, Santa Clara, CA, USA). All workstations were the same for easier maintenance, compatibility, and interchangeability of workplaces. Each workstation also contained a Logitech BRIO 4K UHD webcam (Logitech International, Lausanne, Switzerland) and a Jabra Evolve 40 headset with a microphone (GN Group, Ballerup, Denmark). Both the webcam and microphone aimed to facilitate networking between pathologists from the same hospital, from different hospitals within the ICS, or even with professionals outside our network.

**Figure 6:**
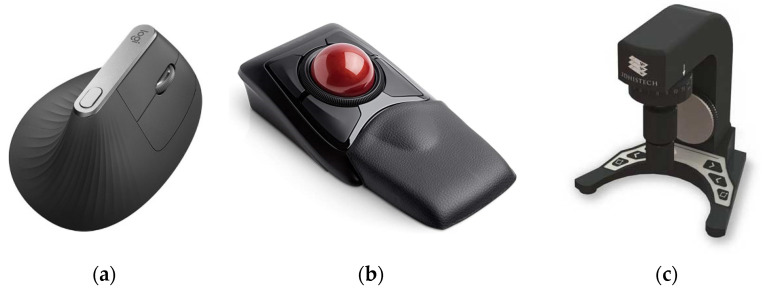
Ergonomic devices: (**a**) Logitech MX Vertical Ergonomic Wireless Mouse; (**b**) Kensington Expert Mouse Wireless Trackball^®^; (**c**) SlideDriver.

Furthermore, each workstation contained a Logitech MX Vertical Ergonomic Wireless Mouse (Logitech International, Lausanne, Switzerland), since vertical mice seem to put less strain on the wrist and demonstrate better ratings than conventional mice [38]. Each pathologist could also choose between two other ergonomic devices: a Kensington Expert Mouse Wireless Trackball^®^ (Kensington Computer Products Group, Redwood Shores, CA, USA) and a SlideDriver (3DHISTECH Ltd., Budapest, Hungary) (Figure 6). All devices were supported by our viewing software. The SlideDriver offers microscope-like navigation on digital slides for those who prefer a traditional method. Most of our pathologists and residents selected the SlideDriver as their input device (80% approximately).

### 2.5. WSI Viewing: Software

**Figure 7:**
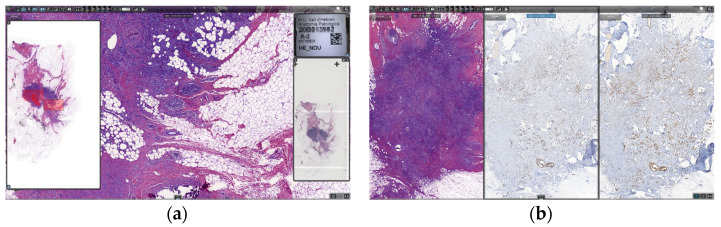
ClinicalViewer: (**a**) slide with navigation panel; and (**b**) three auto-aligned slides.

The diagnostic viewer used for all digital images was ClinicalViewer (3DHISTECH Ltd., Budapest, Hungary). It uses streaming technology to avoid downloading WSIs for diagnosis. It is capable of opening bright-field, fluorescence, double-slide, Z-stack, and macroscopic photography, etc. It also includes many positively valued features, such as the possibility of viewing and navigating up to nine automatically synchronized images at once. It also has IVD support and quantification algorithms, as well as some more standard tools, such as free rotation, free zoom, annotation, measuring, and object counting tools (Figure 7).

The software also had a telepathology option available and a module to create training courses. Possessing all the WSI viewing needs, including fluorescence, integrated into one software system (one viewer) facilitated pathologist ergonomics and also enabled simplifying the technological model with fewer integrations required.

### 2.6. ETPAT: Our Laboratory Information System (LIS)

The evolution of information systems in the last ten years has been meteoric. It has changed the paradigms for accessing and possessing the necessary information at each point of contact a patient has with a health system.

At the Catalan Health Institute (ICS) and within the ARGOS project, we have spent 15 years directing information towards users and clinical care, and we have progressively moved from the initial free texts to structured information. The ARGOS project started in 2006. It is a project to integrate in a transversal and transparent way all the information systems involved in clinical assistance to the citizens of the Catalan Health Institute and its eight hospitals, including the hospital information system, nursing, pharmacy, clinical analysis laboratory, pathology laboratory, and critical care units. Currently, ARGOS is the priority information system in Catalonia and is present in 23 hospitals of the Catalan public health network.

The SALUT4D project began in 2020 and is the evolution of clinical workstations within the ARGOS project. Its objective is to provide the necessary information at each moment of care to different professionals. It is based on four dimensions seeking to present the necessary data at each point of care:

1. Where am I? Scope of work: emergencies, hospitalization, ambulatory consultation, operating room, etc.;
2. Who am I? Nurse, surgeon, internist, psychologist, etc.;
3. Whom do I attend? A patient with hypertension, diabetes, bronchitis, etc.;
4. How do I attend to it? With a computer, tablet, smartphone, etc.

The system presents the information that a professional needs clearly and orderly. The system is based on a clinical dictionary with more than 40,000 variables stored in a MongoDB-type database called the Global Variables Repository (RGV). This repository contains data from all sources: laboratory, pathological anatomy, radiology, vital sign monitors, pharmacy, etc.

To construct this platform, created and designed for and by the ICS, HTML5 technology, SAP^®^ ISH^®^ (Information System Hospital), and SAP^®^ ISHMed^®^ (SAP SE, Walldorf, Germany) were used. The system connects directly with the Shared Clinical History of Catalonia (HC3) and provides access to information on all public hospitals and all primary care facilities in a fully integrated manner.

The work methodology was based on requirements submitted by over three hundred health professionals who continuously provided their contributions. Through AGILE methodologies, the new clinical workstation was progressively built.

The new station is already in operation at the eight ICS hospitals and will gradually come into operation in twenty-three sections of the ARGOS system throughout 2022.

**Figure 8:**
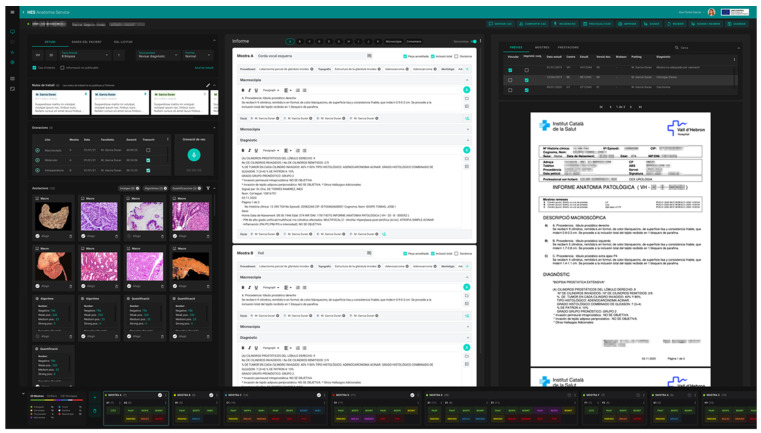
ETPAT screenshot (our proprietary LIS).

In order to incorporate the peculiarities of the new, fully digital workflow, a new LIS for our pathology laboratories named ETPAT was developed. It was deemed necessary to develop and improve pathologist workstations to provide them with tools that allow the integration of information from all sources and electronic records available. ETPAT has a fully integrative approach to all processes occurring in our pathology labs, from electronic requests from clinicians to pathology reports, with full traceability of all steps involved at the laboratory. Moreover, it enables the optimization of paper management as much as possible. This software was based on HTML5 and was prepared for 32-inch 4K UHD monitors, taking advantage of the hardware on hand (Figure 8).

One of the most relevant aspects was the unification of workflow for all ICS laboratories, which should be able to work seamlessly as a network, or even as one big institution. The catalogs of techniques and sample types were also more coherent. All traceability data was integrated and convenient, and we were able to collect information on who carried out each phase of the diagnostic process to allow the correct attribution of costs and activities.

This new LIS is clearly ready for the digital workflow of macroscopic and microscopic images and was fully integrated with the image management platform. In addition, the management of second opinions and case consultations was incorporated.

### 2.7. Network Needs, Local Area Network (LAN), and Wide Area Network (WAN)

The main source for generating information by volume of data was the scanners (in the order of around 2 petabytes yearly), which were located across the different centers included in the scope of the project.

The systems were connected to a 10 Gbps network between the scanners and the local storage data processing center (DPC). The same criteria applied to the artificial intelligence platform.

The network requirements for the DigiPatICS project were high, since access to the images needed to be instantaneous, as it was on a microscope. Images were accessed via streaming, but a large bandwidth was still required, making it necessary to adapt cabling and infrastructure, as well as switches, routers, and other telecommunications equipment. Each workstation required a 300 Mbps connection to the DPC to be able to stream the WSIs, but to ensure performance, the connection was established at 1 Gbps. MacroPATHs required a 100 Mbps connection.

Adjustments were made to the WAN infrastructure to ensure that communications between the central repository and the centers were capable, on one hand, of transferring daily information to the central DPC and, on the other hand, of allowing consultation between hospitals and their corresponding DPCs. This WAN needed to support an approximate flow of 6 TB each night for the DigiPatICS project activity alone.

To achieve these goals, both the Department of Health and Catsalut public institutions recognized this need and facilitated the creation of a dedicated network to support the data traffic requirements mentioned and response times for data recovery.

### 2.8. On-Premise Data Processing Centers (DPCs)

The project proposal was to provide a good user experience to all users of the image management platform, as well as to facilitate their work, optimize performance times, and reduce the time invested in obtaining a correct diagnosis. That is why we considered infrastructure to be a fundamental technological pillar in this project that needed to be sized based on performance, scalability, and reliability criteria without excessively oversizing the system or implementing complex architectures that would create greater difficulties in maintenance. For the project, the deployment of its own infrastructure was carried out, but at the same time, it was integrated transversally across the health ecosystem.

A common structure was used at all centers that only changed in terms of local storage dimensioning, adapting to the number of slides estimated per center. In this way, we made infrastructure maintenance easier. All the DPCs were provided with the energy and cooling requirements necessary for correct operation of the various systems, as well as redundant systems in terms of communications, servers, storage, and a secondary DPC in case of disaster recovery.

The system at each center was designed to have a high-availability configuration, meaning, in the event of a disaster in one of the elements of the system, it would be capable of continuing to offer service with minimum downtime, or even without interruption, depending on the source of the problem.

The high-availability elements implemented were:

- A host server cluster under VMware vSphere in an active-passive configuration;
- Disk storage configured in RAID;
- Mirrored disk arrays with asynchronous replication.

The various redundant elements of the high-availability scenario were located in a DPC or complementary technical room that was not the main DPC in order to minimize the impact of any possible disaster on the main DPC. Artificial intelligence equipment was excluded from this high-availability policy.

At each center, a virtualization infrastructure was deployed, consisting of a set of two identical servers under a VMware vSphere infrastructure. This allowed the system to have the capacity to maintain service quality in an event where it was necessary to activate a contingency scenario (disaster recovery) in a matter of a few minutes. These servers were HPE ProLiant DL325 models dimensioned with AMD EPYC processors that offered the necessary computing capacity for all DICOM image integration, storage management, and processing operations that were carried out continuously. They also served as a virtualization platform for the controller structure of the AI cluster.

As for storage, MSA 1050 cabinets were chosen and presented to the host servers through iSCSI controllers. These storage cabinets contained a battery of SSD disks intended exclusively for virtual machine operating system disks. For medical image storage, a pool of rotational HDD disks was deployed. This decision was made based on the following factors:

- Once the medical image was generated, it was not modified (writing on the disc only one time);
- Access was completed in blocks through streaming (reduced reading rate);
- Each center had its own local storage (limited number of simultaneous writes).

For all these reasons, the increase in cost of deploying SSD technology for image storage was not justified for the required performance.

The same storage technology was used in all the centers, including the central DPC, with only capacity varying as a factor of the number of digitized images estimated per center. The total storage capacity between the eight hospitals was approximately 500 TB, which allowed short-term storage of up to 75 days for images.

The deployed server and storage infrastructure had the activation of the HPE IRS service, which automatically opened a ticket to the manufacturer in the event of a hardware component failure, guaranteeing proper functioning of the infrastructure.

The entire server infrastructure was backed by a backup policy based on Veeam Backup that was integrated into existing control panels in each center in order to facilitate maintenance tasks.

Within the scope of this project, integration with SIMDCAT was included for the publication of images as a form of definitive storage. Publishing to SIMDCAT was asynchronous under a queue management system that published the images as soon as they became ready. As the publication to SIMDCAT was continuous, the need to make a backup copy of all the scanned images outside the same disk array cluster was not considered, delegating the backup function to the same SIMDCAT. If the need arose to retrieve one or more images, a small application was implemented that allowed downloading these images from SIMDCAT and placing them back in local storage. Only in the event of a major disaster would the procedure be the same as indicated above; however, there may be a minimal number of scanned images that would not have had the opportunity to be published in SIMDCAT due to the asynchronous nature of the publication process. In such a case, the images would be rescanned.

### 2.9. SIMDCAT and DICOM

SIMDCAT is the Digital Medical Image System of Catalonia. It is currently the unified system used by the network of entities called SISCAT (Comprehensive Health System of the Public Utility of Catalonia) to preserve digital medical images and provide services and digital resources based on the same software architecture using cloud services and to make information accessible from various electronic medical records. The system is used by approximately 450 Catalan health centers and by all SISCAT professionals to securely collect and share digital medical images generated by the centers. SIMDCAT was the definitive repository of this project in which the images and associated data were stored. SIMDCAT was set up in 2018 to provide the public health system with a secure and technologically advanced environment in which to store and share digital medical image services.

The project to develop in SIMDCAT a specific environment for pathological anatomy medical images presented multiple benefits for agents of the Catalan health system. Professionals in this specialty were provided with a network work environment based on cloud technology, and the anatomopathological diagnosis process was optimized so that medical images were immediately and safely available to professionals. As for patients, the quality of care was improved, and their safety was increased.

Finally, the sustainability of resources and costs of health information systems was promoted by providing a common and shared system for all providers.

SIMDCAT’s cloud-based technology is focused on the use of DICOM as a standard in the storage and distribution of medical objects. The capacity of standard DICOM beyond radiology has been demonstrated in recent years, where PACS image storage systems have evolved into the VNA (Vendor Neutral Archive); SIMDCAT constitutes a VNA that allows the management of medical objects using the DICOM standard as a reference framework. In the case of pathology, the DICOM 145 extension is adopted; this extension facilitates the storage and distribution of medical objects independent of the device that generates the information. SIMDCAT adopts DICOM 145 natively, incorporating the pyramidal treatment of images, thus facilitating navigation through an image. SIMDCAT also adopts the DICOM 122 extension that reflects the representative data model of pathology.

SIMDCAT manages the information in a private cloud model with a high-redundancy system to guarantee the availability of the information. Saving the information in different storage systems using data object technology, this model allows growth according to the needs of the system, optimizing available resources in addition to freeing hospitals from the custody and distribution of information.

### 2.10. Artificial Intelligence and the Polytechnic University of Catalonia–BarcelonaTech (UPC)

It is now recognized that artificial intelligence represents a turning point for society that is at least as significant as the Industrial Revolution. The ICS aims to be an autonomous and primary player in the creation of artificial intelligence algorithms to avoid dependance on a commercial solution. Therefore, we wished to set up an artificial intelligence platform tailored to the needs of the ICS. This platform was developed with free software and needed to be modeled to accommodate other artificial intelligence projects in addition to DigiPatICS.

Currently, the ICS has signed a collaboration agreement with the Image Processing Group (GPI) of the Polytechnic University of Catalonia–BarcelonaTECH (UPC) (imatge.upc.edu) for the development of artificial intelligence tools and platforms within the healthcare field, specifically in the field of medical imaging. The group belongs to the Intelligent Data Science and Artificial Intelligence Research Center (IDEAI), which is a hub created at the UPC in 2017 for the development of artificial intelligence. GPI has extensive experience in image processing and the development of artificial intelligence algorithms with a long history in the healthcare field.

GPI develops computer vision and deep learning (DL) tools to tackle WSI analysis tasks in DigiPatICS for stains, such as H&E, HER2, KI67, RE, and RP, as well as other immunohistochemical stains. DL technology that relies on instance and semantic segmentation architectures has the potential to provide high-quality results when properly trained. GPI has also introduced strategies, such as:

**Dataset annotation:** As the training database was limited in the first stage, a more classical computer vision strategy relying on morphological algorithms and machine learning (ML) tools produced proposals easier to validate by annotators and generated the ground truth needed to avoid limiting the performances of DL approaches.

**Integration of AI algorithms:** Integration into specialist workflow was facilitated by combining the systematic processing of a large number of WSIs with on-demand assessment by pathologists for improving the systematic results or for obtaining specific quantifications:

1. Nightly batch processing and inference on the WSIs yielded raw results, such as segmentation confidences and classification probabilities, as well as potential segmentation masks;
2. The results were integrated into 3DHISTECH ClinicalViewer using a specific plug-in and offered to the pathologists upon request when examining the slides;
3. The pathologists could select or deselect regions in the WSI to visualize and quantify the results or to fine-tune inference results (classification and segmentation) using sliders;
4. Pathologists could also select specific areas for further online analysis on inference servers to be performed during the session with the viewer at their workstations.

**Pathologists in the analysis loop:** The strategy in Point 3 allowed not only flexible interaction for online and on-demand analysis, but also for recovering information about pathologists by selecting specific regions of interest and tuning inference results for reports. This information represents invaluable data and comments to further improve the annotated datasets.

**Training in the ICS development servers:** A specific committee formed by AI specialists and clinicians periodically reviewed the comments and data produced by pathologists using the viewer. This committee decided on the feedback and data and set strategies for incremental training and improvement of the DL network architecture involved with continuously improving the inference results.

The former strategies facilitated the usage of AI tools in the daily work of pathologists, as well as productivity.

As mentioned, all images were stored in a central repository, SIMDCAT, where they were available for AI training after dissociation. AI training was performed in this central repository, where a large number of whole slide images were readily at hand. GPI researchers did not have direct access to sensitive clinical data, and datasets and whole slide images always remained within the ICS infrastructure. In fact, it should be noted that the entire circuit in production moved through an isolated dedicated network in an intranet environment, and the training environment was in a separate VLAN and intranet infrastructure. Once the inference algorithms were trained, they were run on-premise at each hospital DPC. In no case was the information transferred to external DPCs.

## 3. Results

The digital pathology transformation envisioned in the DigiPatICS project involved great changes in most of the steps of workflow in our pathology laboratories. As mentioned above, all the processes were integrated in our LIS ETPAT.

### 3.1. Laboratory Workflow, Traceability, and Barcode Generation

All incoming samples, biopsies, cytologies, autopsies, and even molecular requests are accompanied by an e-request generated in our electronic health record system by the clinicians who input all the necessary clinical data.

**Figure 9:**
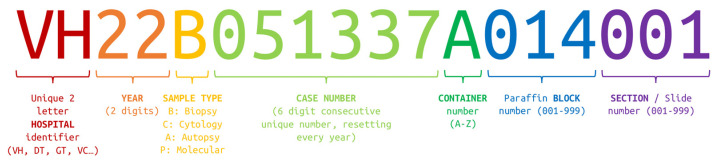
Barcode anatomy.

At the time of registration, the request receives an identification number that is labeled with a datamatrix-type barcode. Each sample container is also labeled with a unique datamatrix-type barcode. The barcode structure is as follows, maintaining logical hierarchy, as seen in Figure 9. The case or request barcode includes the institution, the year, the sample type, and the case number (i.e., VH22B051337, for Vall d’Hebron, year 2022, biopsy number 51,337). When labeling a container, the container ID is appended (i.e., VH22B051337A for container A). When a cassette is required, a datamatrix-type barcode is also printed with the number of the paraffin block appended (i.e., VH22B051337A014 for block 14). For glass slides, whether they come from paraffin block sections or are cytologies, another number is appended, representing the slide number (i.e., VH22B051337A014001 for histological section 1). The full ID is printed and appears in a datamatrix-type barcode in each slide.

Most of our laboratory equipment, including scanners, is able to read these barcodes and is integrated with our LIS, providing automatic checkpoints for many steps of processing.

The first checkpoint is when the sample is received with the labeling of the containers, as described previously. It is a manual step completed by technicians, but in the new LIS, the process was simplified so it was closer to optimal. The next step, grossing, was also improved: MacroPATHs were installed inside the pathology fume hoods, and computers with the LIS were placed next to them. Pathologists, residents, or technicians could print the cassettes, capture gross examination pictures, and dictate or type the macroscopic description in a convenient and fully digital way. Pictures of cassette contents are obtained and are available for verification in the next laboratory steps. After filling a cassette with tissue, the barcode is scanned for the first checkpoint of the paraffin block. The next checkpoint is when the cassette enters the tissue processor, and a technician reads the barcodes using a handheld device. Unfortunately, this step remained manual because our tissue processors could not read barcodes. We hope this will change in the future.

Each embedding station had a computer with a barcode reader within reach. A technician read the barcode of the paraffin block, and the LIS displayed the tissue type and the confection notes (if present) and provided access to macroscopic images for verification, while, in fact, creating another checkpoint.

The next checkpoint is clearly when sectioning. Every microtome station had a barcode reader, a slide label printer, and a computer with the LIS. When scanning the paraffin block, the LIS displayed the tissue type and other information on that block, such as how many and which slides must be obtained from it. At the same time, it printed the slide labels automatically, which include the datamatrix barcode described previously. This automation reduced the human error of manual workflow [3].

At this point, the automatic part started. In most hospitals, Sakura Tissue-Tek Prisma Plus and Tissue-Tek Film (Sakura Finetek Europe B.V., Alphen aan den Rijn, The Netherlands) were used. After sectioning, a technician loaded the slides in the Sakura racks. Then, the racks were stained, coverslipped with film, dried, and scanned using P1000s. Routine slides did not have to be treated individually until archiving because all the equipment used the same racks. In terms of traceability, an automatic checkpoint was recorded by Sakura Tissue-Tek Film, which was integrated with our LIS, and the final checkpoint was notified by a 3DHISTECH P1000 when the slide was scanned.

Using the same Sakura racks for all equipment enabled us to optimize technician slide handling time and reduced errors. Immunohistochemistry and histochemistry used the same Tissue-Tek Film, so checkpoints stayed the same. In cases of double slides, the steps needed to be conducted manually, but their volume was negligible compared to routine histology, where workflow optimization was key.

In some hospitals, other automatic stainers were used, such as VENTANA HE 600 (Roche, Basel, Switzerland). Integration was possible, but an extra step of transferring the slides from HE 600 trays to the scanner rack was needed.

Subprocesses, such as slide allocation and delivery to pathologists, did not exist anymore, since the digital workflow did not require such manual steps.

Regarding the destruction of containers, a new system was implemented. A tablet with the LIS, along with a handheld datamatrix barcode reader, was used to scan the containers. If the containers were ready for elimination, a confirmation message appeared, and a checkpoint was created.

The whole workflow was designed for optimization and security. We aimed to reduce the time technicians spent on worthless tasks, allowing them to focus on what was important and increase their satisfaction in the workplace. Also, the checkpoints enabled a better awareness of sample status by all personnel and an obvious increase in safety.

It is also worth mentioning that the transformation was carried out simultaneously as a radical change with all the pathologists in the same center starting digital work at the same time. The alternative of making a gradual change implied many more difficulties by having to maintain two circuits (old and new) in the same laboratory. This measure was taken considering the possible reluctance and skepticism of the staff, which would be lessened if they could quickly see an optimization of circuits despite initial incidents. No benefits of a gradual approach were seen by the authors if all the infrastructure was in place.

Regarding validation of WSIs for use in primary diagnosis, the digital pathology solution provided all necessary legal certifications, but it still was tested and validated previously by many pathologists using all types of preparations. The solution was also tested in the scanner and viewer technical tests during the bidding process. When the pathologists started working with digital slides, all of them could compare digital slides with glass slides until their grade of confidence was enough. No specific period of time or amount of resources was dedicated for this purpose; it was a matter of obtaining enough comfort for the pathologists in their routine workflow. A continuous validation of tissue detection was performed in every single case, since the pathologists had available the captured image of the slide next to the slide overview, as seen in Figure 7a, and were thus certain there was no tissue missing on the WSI.

Furthermore, all the pathology departments involved in DigiPatICS were certified or accredited for ISO 9001 or ISO 15189. Because the ISO 15189 standard confirms technical competence of the laboratory and ensures reliability of test results, a continuous validation strategy was recommended following CAP recommendations [39,40].

The initial reticence on the part of a couple pathologists could not be reversed despite several talks and explanations to try to make them partners in the transforming project. However, these reluctances completely disappeared after a few days of working in the digital flow without the need for any external intervention. The advantages of the new technology were obvious and sufficient on their own.

### 3.2. Scanning

Being able to scan slides and obtain WSIs are perhaps the obvious concerns of any digital pathology project, but, as mentioned, these are not the only relevant points.

It was essential that the scanners did not stop due to jamming, so the glass slides needed to be optimal. This implied possessing a decent glass or film coverslip; either could work as long as the quality of the histological slide was good. Labels also needed to fit perfectly within the boundaries of the glass slide. Anything that protruded could cause jamming. Slides needed to be fully dried, and under no circumstance could be dripping glue [20].

Furthermore, we considered crucial for the workflow the presence of automatic scanning with two objectives and, therefore, two resolutions, 40× (0.25 μm/pixel) and 80× (0.12 μm/pixel), to cover all possible needs. The scanners used different scanning profiles (40× or 80×, Z-stack, etc.) depending on the sample and the stain type indicated by our LIS (ETPAT). The process was fully automatic. It was also possible to select profiles manually or tweak some settings, but manual parameterization was not used for high-throughput routine scanning. Routine scanner loading needed to be as easy, fast, and straightforward as possible for the laboratory technicians. In order to have the automatic profile feature in addition to automatic tissue detection obtained by an AI algorithm in the scanner control software, it was very important to scan slides at a suitable resolution.

The second concern regarding scanners was their deployment. Correct dimensioning is essential to address daily routine activity. Individual scanning speed or capacity is very important, but less relevant than the deployment of equipment meeting the needs of the center. In the event of a breakdown or maintenance, the remaining equipment must be able to compensate. Scanner deployment must also take into account the expected growth of activity (in our case, 10–15% annually). Scanners must not create bottlenecks in the workflow, and going fully digital cannot involve a delay in turnaround time. In our daily routine, scanning occurred right after the slides were dried, and the first WSIs were available during the morning. The scanners were continuously loaded until the end of the technician shift for each institution (in some cases 16:00, and 21:00 in others), and the scanners were supposed to finish scanning overnight, around 2:00–3:00 a.m. in our institutions producing more slides, thus having time until 8:00 a.m. to accommodate this expected increase in activity before the pathologists started diagnosing. This workflow guaranteed no delay in turnaround time, even after adding steps to the conventional system, by completing slide production and digitation of all slides within the same day. The remaining advantages of digital pathology should optimize our routines and reduce diagnostic and reporting times.

Another aspect considered was where to physically locate the scanners. The location needed to be convenient for technicians within their workflow so that the new steps were not disruptive. Scanners were located near a stainer or coverslipper or next to a glass slide archive whenever possible.

Fluorescence workflow was taken into account to try to keep the slides cool and to preserve them from light.

### 3.3. Monitors

The need for a high-quality monitor is indisputable, although recommendations for ideal screen size and resolution have changed over time. Currently, a size between 24 and 32 inches with a high resolution is considered necessary, and the trend is probably upwards. A larger monitor, such as the ones we used, with a smaller pixel size allows for a greater field of vision, avoiding displacement through digital preparation. However, this means that objects appear smaller when the original maximum magnification is reached and that it requires more bandwidth to stream the image [41,42,43].

**Figure 10:**
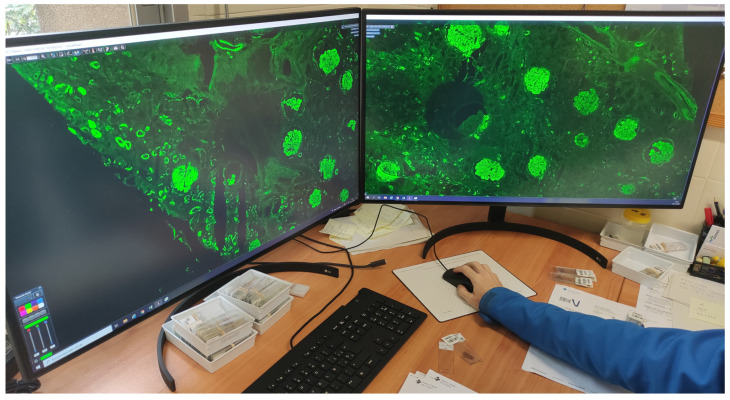
Fluorescence seen in a totally integrated fashion on two 32-inch LG 32HL512D 8MP diagnostic monitors.

It is also important to take into account color fidelity (the panel can be calibrated), lighting (no backlight bleed), contrast, pixel size, pixel density, brightness, color space (sRGB, Adobe RGB), color depth, etc. However, a good monitor is not going to make up for poor digital preparation. A high-quality monitor is important to guarantee image fidelity and ergonomics, as well as to avoid visual fatigue for the user (Figure 10).

### 3.4. Teaching, Telepathology, and Networking

Many of the planned advantages of this project, such as networking between ICS hospitals, having a teaching platform, and being able to teleconsult cases with external pathologists, although not available right now, were binding in the contract and will be implemented in the following months [44]. The system will have an on-demand function to publish studies to an Internet environment with previously anonymized data to allow the sharing of images.

## 4. Discussion

The DigiPatICS project, as a transformation of the Catalan Health Institute Network of eight hospitals, represented an important technological, organizational, and functional challenge.

We incorporated digital pathology and artificial intelligence in pathology departments with an organizational change that modified work dynamics, as seen in the previous paragraphs. This change responded to current and future challenges with the aim of improving quality, efficiency, effectiveness, equity, speed, systematization, and reproducibility of diagnoses. These improvements affected other disciplines and increased patient safety with the following benefits: improvement of diagnostic conditions by incorporating the digitalization of preparations with maximum guarantees of traceability while minimizing material losses and identification errors; and improvement of workflow, productivity, and turnaround times. Moreover, there was also improvement in working conditions in terms of ergonomics. The viewers also provided tools for morphometry and quantification of diagnostic and prognostic biomarkers and facilitated the digital access of preparations from previous patient examinations without interrupting the diagnostic process [4,7].

In the near future, we will enable access to images for decision making between pathology departments and other facilities (operating rooms, interventional examination rooms, transplantation, or clinical committees) using bidirectional communication. Also, the images will be available for clinical sessions, tumor boards, and pre- and postgraduate teaching. Being able to store pathology images digitally also reduces the workload for management of histological preparation files. Ergonomics, workflow improvement, and convenience of access to historical slides also encourage research.

Successfully exchanging whole slide images and artificial intelligence algorithms between institutions had the following implications: We improved the reproducibility of diagnoses, both in terms of interpretation and in the way they are reflected in medical reports, ensuring system-wide equity and fairness. We encouraged an optimal exchange of information between hospitals, establishing second-opinion strategies according to clinical practice guidelines that immediately benefitted patients. Thus, we also encouraged the movement of patients between hospitals in a coordinated way, avoiding the physical movement of biological material with fewer delays and courier costs and ensuring preservation. Lastly, we aimed to make more efficient use of the existing critical mass in terms of prevalent, complex, infrequent, and difficult diseases and guaranteeing equity between hospitals, regardless of size and geographical location, with the end-goal of organizing references for pathologies and territories.

From a technological standpoint, innovation also occurred. We stored all images using DICOM standards in SIMDCAT, a unified system used by the SISCAT network of entities to preserve digital medical images, provide digital services and resources based on the same software architecture, and make digital medical images accessible. This repository can be used independently by different viewers and is also independent of the scanning system. The transformation of the laboratory information system and all IT infrastructure is key in this type of project.

In addition, from the technological point of view, we aimed to achieve an improvement in algorithms for the quantification of immunohistochemical biomarkers and for the assessment of in situ hybridization. In the future, we hope to develop artificial intelligence algorithms with machine learning and deep learning in order to recognize patterns and segment tumor areas. We need to look for tools that help pathologists do their jobs with AI algorithms developed by our researchers. We produced an image repository large enough for this aim. Artificial intelligence optimizes the reproducibility of diagnoses. The boom in artificial intelligence will involve very significant changes in the way pathologists work in the coming years.

All things considered, it is important to seek a digital pathology system and, therefore, a manufacturer and a distributor that adapt to the real needs of each particular case. In the DigiPatICS project, we looked for a complete holistic solution for our pathology departments. As shown, it was very difficult to compare products according to data sheets exclusively, and it was very laborious to define a digital pathology project for all technical and functional implications.

The growing enthusiasm for digital pathology and the new possibilities artificial intelligence offers indicate an emerging revolution in pathology that will change our way of working. However, for broad adoption, an integrative approach of digital pathology across clinicians, pathologists, laboratory information systems, viewers, hardware, research, and teaching is imperative. Digital pathology must simplify our workflows and not add complexity. Vast repositories of diagnosed images will allow us to make great strides in this direction. With our solution meeting these needs, we hope to inspire other pathologists and to provide useful guidance for their successful digital transformations.

## 5. Conclusions

The DigiPatICS project aimed to deploy digital pathology in an integrative, holistic, and comprehensive way within a network of 8 hospitals, incorporating 168 pathologists and over 1 million slides each year. After careful planning, implementation was carried out simultaneously for all the pathologists in each institution. A digital pathology system needed to be integrated with all health information systems, including electronic medical records. Teleconsultation, teaching platforms, fluorescence, and cytology were taken into account. The digital transformation of a pathology department represented a technological, organizational, and functional challenge. It provided an effective and safe diagnostic tool with clear benefits for diagnosis quality and patient safety.

## Figures and Tables

**Table 1:** Number of slides in 2019.

Routine Histopathology	814,573
Immunohistochemistry	186,453
Histochemistry	64,209
Direct Immunofluorescence	12,392
FISH	2695
CISH	1983
**Total**	**1,082,305**

**Table 2:** Scanner deployment and capabilities summary.

Model	HT	DS	FL	IN	Z	S	N
PANNORAMIC 1000 Flash DX	✓	✓		✓	✓	1000	**11**
PANNORAMIC 300 Flash DX	✓		✓		✓	300	**7**
PANNORAMIC SCAN II			✓		✓	150	**5**
PANNORAMIC MIDI			✓		✓	12	**1**
**Total**							**24**

**HT**: high throughput capability; **DS**: double-width slide capability; **FL**: fluorescent scanning capability; **IN**: immersion scanning capability; **Z**: Z-stack scanning capability; **S**: slide capacity; **N**: number of deployed scanners; ✓: Capabilities available in each scanner.

**Table 3:** Workstation and monitor deployment.

Equipment	N
Workstations	183
32″ 8MP Medical Monitor LG 32HL512D	286
55″ 4K UHD Monitor 55UH5F-B	6
4K UHD DICOM ProBeam LG Projector	7

## Data Availability

All data generated or analyzed during this study are included in the article.
